# Novel link between plasma bilirubin and anti-inflammatory miRNA profiles in follicular fluid of IVF patients

**DOI:** 10.1152/ajpendo.00479.2024

**Published:** 2025-05-05

**Authors:** Natascha Berger, Anna Rieder, Katharina Brugger, Bettina Amtmann, Martina Kollmann, Irmgard Oreskovic, Slave Trajanoski, Ursula Hiden, Herbert Fluhr

**Affiliations:** 1 Department of Obstetrics & Gynecology, Medical University of Graz, Graz, Austria; 2 Department of Biomedical Science, Carinthia University of Applied Sciences, Klagenfurt, Austria; 3 Division of Physiology and Pathophysiology, Cardio-Metabolic Research Group, Medical University of Graz, Graz, Austria; 4 Core Facility Computational Bioanalytics, Medical University of Graz, Graz, Austria; 5 Research Unit Early Life Determinants (ELiD), Medical University of Graz, Graz, Austria

**Keywords:** Bilirubin, female metabolism, fertility, follicular fluid, IVF, miRNA

## Abstract

Maternal metabolic factors are increasingly recognized as critical pre-conceptional determinants of fertility outcomes. To investigate how metabolic health influences female fertility, we investigated the molecular composition of follicular fluid (FF), with a focus on microRNA (miRNA) expression. Blood and FF samples from 15 women undergoing controlled ovarian stimulation were examined in a pilot study. Clinical traits (glucometabolic markers, lipid profiles, liver function markers, inflammatory cytokines, and hormonal parameters) and oocyte outcomes were measured and recorded. Elevated plasma bilirubin levels were associated with a distinct miRNA profile in FF, characterized by an enrichment of anti-inflammatory miRNAs, including miR-146a-5p, miR-146b-5p, miR-487b-3p, and miR-21-5p. Bioinformatic analysis revealed that these miRNAs directly target key inflammatory mediators, including IL6, COX2, TLR4, IRAK1, and NFKB1, suggesting a regulatory role in intra-follicular inflammation. Furthermore, patients with a fertilization rate of ≤50% exhibited higher transcript levels of miRNAs associated with elevated plasma bilirubin. Our findings provide a novel perspective on the growing body of evidence supporting bilirubin’s regulatory properties, including anti-oxidative and anti-inflammatory effects and highlight the relationship between plasma bilirubin and FF miRNA expression. The observed associations between bilirubin levels, follicular fluid miRNA composition, and oocyte quality underscore the critical influence of metabolic factors on reproductive outcomes. This exploratory work provides a foundation for further studies to investigate the functional role of plasma bilirubin in follicular physiology and its potential as a biomarker to optimize fertility treatments.

## Introduction

Infertility is increasingly evolving into a global burden, affecting 8-12% of women during their reproductive age (1). The rising demand for reproductive medical measures, such as *in vitro* fertilization (IVF) and intracytoplasmic sperm injection, is also illustrated by the high number of more than 1 million treatment cycles reported from 40 European countries in 2019 (2). The success rates of assisted reproductive technologies (ART) are attributed to various etiologies of infertility. In addition to parental reproductive aging, certain clinical factors such as polycystic ovary syndrome (PCOS), endometriosis or tubal disease are the most common indicators for an IVF treatment (3). Moreover, approximately 15% of infertile couples are diagnosed with ‘unexplained infertility’ (4). Accumulating evidence indicates that the metabolic status of women is a key factor associated with reproductive health outcomes (5). Since the prevalence of obesity and associated metabolic disturbances, such as low-grade chronic inflammation and type 2 diabetes mellitus, is predicted to increase in the future (6), a comprehensive approach to assess metabolic risk factors is highly relevant to improve infertility procedures in an individualized manner.

MicroRNAs (miRNAs) are small, non-coding, evolutionarily highly conserved, single-stranded RNA molecules, that function in a highly coordinated manner to precisely control protein expression. They target messenger RNAs via sequence-specific base pairing, resulting in mRNA degradation or repression of translation. The critical role of miRNAs in regulating biological functions is gaining significant recognition, with numerous miRNAs currently being investigated as biomarkers in clinical trials and others being explored as potential therapeutic targets (7). Moreover, the most recent release of the miRNA database miRBase (v22.1) catalogs more than 2,600 mature human miRNAs. Of these, around 600 are annotated with high confidence, verified for both sequence and function (8).

In female reproduction, miRNAs play crucial roles by regulating cell differentiation, cell cycle events, and immune responses (9,10). Research has highlighted their involvement in key reproductive processes such as oocyte maturation, follicular development, and hormone production, directly affecting fertility outcomes and embryo quality (11). Furthermore, dysregulation of specific miRNAs has been associated with infertility and a range of reproductive disorders, including PCOS, endometriosis, and premature ovarian failure (12–15).

This exploratory study aims to investigate the influence of maternal metabolic factors — encompassing glucometabolic profiles, lipid metabolism, liver function, hormonal and energy regulation, and inflammatory markers — on the intrafollicular milieu, with a particular focus on miRNA expression. As FF miRNAs play a pivotal role in reproductive events, and pre-conceptional metabolic risk factors are increasingly recognized for their impact on fertility, we hypothesize that these factors may collectively impact IVF outcomes. In the present study, we seek to gain new insights on the impact of metabolic risk factors on reproductive outcomes and their potential implications for clinical practice in fertility treatments.

## Materials and Methods

### Study design and subject characteristics

Recruitment of women undergoing ART took place at the Division of Gynecological Endocrinology and Reproductive Medicine at the Medical University of Graz during routine visits in the outpatient clinics. This investigation was prospective and observational and received approval from the Ethical Committee of the Medical University of Graz (Approval No. 34-459 ex 21/22). All participants gave written informed consent. For miRNA sequencing a pilot cohort including a total of 25 FF samples was analyzed. Subject baseline characteristics are outlined in Table 1. Study participants exhibited a broad representation of key variables, including a wide range of BMI, body composition parameters such as fat and fat-free mass, and age. Individual body composition, including fat and fat-free mass, was assessed using air displacement plethysmography (BOD POD^®^). While these samples were selected to reflect a diverse physiological spectrum, metabolic health including insulin sensitivity, liver function, hormonal status and clinical diagnoses of infertility and underlying comorbidities were matched to ensure consistency in disease states across the cohort (Supplementary Figure 1A). Additionally, none of the patients had a clinical diagnosis of metabolic disorders such as type 2 diabetes mellitus or Gilbert’s syndrome. Women with PCOS were excluded from this study due to the considerable heterogeneity associated with the condition. PCOS presents a wide range of clinical manifestations, including variations in hormonal profiles, metabolic disruptions, and follicular dynamics (16). These complex and highly variable factors could introduce confounding variables that would complicate the interpretation of miRNA expression patterns, especially in a study with a limited cohort size. The untargeted nature of miRNA sequencing does not allow for precise sample size calculations in advance, as the primary aim is hypothesis generation rather than validation.

### Collection of follicular fluid

Depending on the patient’s individual hormonal profile as well as response to stimulation they were either treated with a GnRH agonist protocol including Decapeptyl (0.1 mg/ml, Ferring Pharmaceuticals, Switzerland) and Meriofert (300 IE, IBSA Institut Biochimique SA, Switzerland) or with a GnRH antagonist regimen including Cetrotide (0.25 mg, Merck Serono, Switzerland) or Orgalutran (0.25 mg, Organon, NJ, USA) and recombinant FSH (Ovaleap 150 IE, Theramex, Germany; Gonal F 300/ 250/ 225/ 150 IE, Merck Serono, Switzerland; Rekovelle 12 mg, Ferring Pharmaceuticals, Switzerland) or a combination of FSH and human chorionic gonadotropin (Menopur 300/ 100 IE, Ferring Pharmaceuticals, Switzerland; Meriofert 300/ 225 IE, IBSA Institut Biochimique SA, Switzerland; Pergoveris 150/ 75 IE, Merck Serono, Switzerland) for follicle stimulation. When at least 1 ovarian follicle had grown to 18 mm in diameter, Zivafert (10.000, IBSA Institut Biochimique SA, Switzerland) or Ovitrelle (250 µg/0,5 ml, Merck Serono, Switzerland) was administered, and the follicles were aspirated 36 hours later. FF was sampled by transvaginal ultrasound-guided puncture and carefully collected from the first aspirated follicle from each donor. FF samples were centrifuged at 810 x g for 10 min at 4°C to remove potential contaminants of cells and cell debris. The remaining material was subsequently aliquoted and stored at -80°C until further processing.

### Blood collection

Venous fasting blood samples were collected before follicular puncture procedure using VACUETTE^®^ tubes (Greiner Bio-One GmbH, Kremsmünster, Germany). Serum and plasma fraction was separated by centrifugation and glucometabolic parameters (Insulin, C-peptide), lipid metabolic parameters (cholesterol, high-density lipoprotein-cholesterol (HDL-cholesterol) low-density-lipoprotein-cholesterol (LDL-cholesterol)), parameters of liver function (albumin, aspartate aminotransferase (AST), alanine aminotransferase (ALT), gamma-glutamyltransferase (GGT), alkaline phosphatase (ALP), cholinesterase (CHE), total bilirubin, conjugated bilirubin), hormonal and inflammation markers (thyroid-stimulating hormone (TSH), basal cortisol, C-reactive protein (CRP), anti-mullerian hormone (AMH)) were analyzed at the Department of Obsetrics and Gynaecology and the Clinical Institute for Medical and Chemical Laboratory Diagnosis at the Medical University of Graz following manufacturer protocols. Unconjugated bilirubin was calculated by subtracting the conjugated fraction from total bilirubin. Glycated hemoglobin (HbA1c) and blood counts (erythrocytes, hemoglobin, hematocrit, mean individual erythrocyte volume (MCV), mean corpuscular hemoglobin (MCH), mean corpuscular hemoglobin concentration (MCHC), mean platelet volume (MPV), leukocytes, thrombocytes, lymphocytes, monocytes) were analyzed at the Clinical Institute for Medical and Chemical Laboratory Diagnosis at the Medical University of Graz according to manufacturer protocols using whole blood. Further, IVF parameters including antral follicle count (AFC, <15mm), follicular output rate (FORT), follicle to oocyte index (FOI), oocyte utilization rate, fertilization rate, blastocyst formation rate and embryo quality were measured and recorded at the Division of Gynecological Endocrinology and Reproductive Medicine. Moreover, implantation rates for both fresh and cryopreserved single embryo transfers and live birth rates within one ovarian stimulation cycle were calculated. The live birth rate accounts for all subsequent fresh and frozen embryo transfers derived from a single ovarian stimulation cycle.

$$\begin{array}{c} \;FORT\;=\frac{\;preovulatory\;follicle\;count\;}{\;AFC\;}\\ \;FOI\;=\frac{\;number\;of\;retrieved\;oocytes\;}{\;AFC\;}\\ \;Oocyte\;utilization\;rate\;=\frac{\;MII\;oocytes\;}{\;number\;of\;collected\;oocytes\;}\\ \;Fertilization\;rate\;=\frac{\;number\;of\;oocytes\;with\;two\;pronucle\;i\;}{\;number\;of\;collected\;oocytes\;}\\ \;Blastocyst\;formation\;rate\;=\frac{\;number\;of\;blastocysts\;}{\;number\;of\;oocytes\;with\;two\;pronuclei\;}\\ \;Implantation\;rate\;=\frac{\;number\;of\;gestational\;sacs\;}{\;number\;of\;transferred\;embryos\;}\\ \;Live\;birth\;rate\;=\frac{\;number\;of\;live\;births\;}{\;number\;of\;transferred\;embryos\;} \end{array}$$

### RNA isolation

RNA extraction was performed using miRNeasy Serum/Plasma Advanced Kit (Qiagen, Hilden, Germany). This kit enables total RNA extraction including extracellular vesicular and free miRNA. RNA isolation was performed according to manufacturer protocols. Briefly, for the extraction, 200 µl of FF was mixed with 60 µl of RPL buffer to induce the lysis of exosomes and plasma proteins. An optional on-column DNase digestion using RNase-Free DNase Set (Qiagen, Hilden, Germany) was performed to meet sequencing quality requirements. The quality of the miRNA obtained was determined using the Small RNA Kit on Bioanalyzer (Agilent, CA, USA). Quantification of the extracted RNA was performed using Qubit™ microRNA assay kit (InvitrogenTM, Thermo Fisher Scientific, MA, USA) on the Quantus™ fluorometer (Promega, WI, USA).

### Library preparation and miRNA sequencing

The library preparation was performed using QIAseq^®^ miRNA UDI Library Kit (Qiagen, Hilden, Germany) according to the manufacturer’s instructions. In brief, small RNAs were ligated with 3′ and 5′ adapters, followed by reverse transcription with primers assigning unique molecular indexes to single-stranded cDNA molecules. After miRNA purification, library amplification was performed, assigning unique dual indexes to each reaction. After final purification, library quantification and quality control were performed using QuantiFluor^®^ ONE dsDNA System (Promega, Wisconsin, USA) on Quantus (Promega, Wisconsin, USA). Quality verification was carried out by Bioanalyzer (Agilent, Santa Clara, USA) using the Agilent High Sensitivity DNA Kit. MiRNA libraries peaked around 200 bp. Multiplexed samples (1.2 pM) were sequenced as 75 single-read cycles on a MiniSeq System (illumina, CA, USA). Data analysis was performed on GeneGlobe (Qiagen, Hilden, Germany) using the RNA-seq Analysis & Biomarker Discovery Pipeline. FASTQ files were aligned against the reference database miRBase_v22, Homo sapiens (GRCh38.103) in the RNA-seq Analysis Portal 4.0 to obtain total count sequence reads.

### Sequencing data normalization and over-representation analysis

In total 1054 miRNAs were detected in FF samples. Unsupervised clustering and data normalization was conducted using DESeq2 regularized log transformation (17), which facilitated pre-filtering of low count genes by setting a pre-filter threshold at 100 reads across all samples. This step assured that only miRNAs with sufficient expression levels were included in the analysis, thereby focusing on highly expressed miRNAs. Hence, subsequent statistical assessments were based on non-sparse data, leading to more accurate identification of expression patterns and correlations. Overrepresentation analysis (ORA) was subsequently carried out using the miRNA Enrichment Analysis and Annotation Tool (miEAA) (18) assessing functional enrichment. For this analysis, a subset of miRNAs exhibiting positive correlations with total bilirubin concentrations (correlation coefficient r > 0.5) was selected.

### Background profiling and sample selection

**Figure 1:**
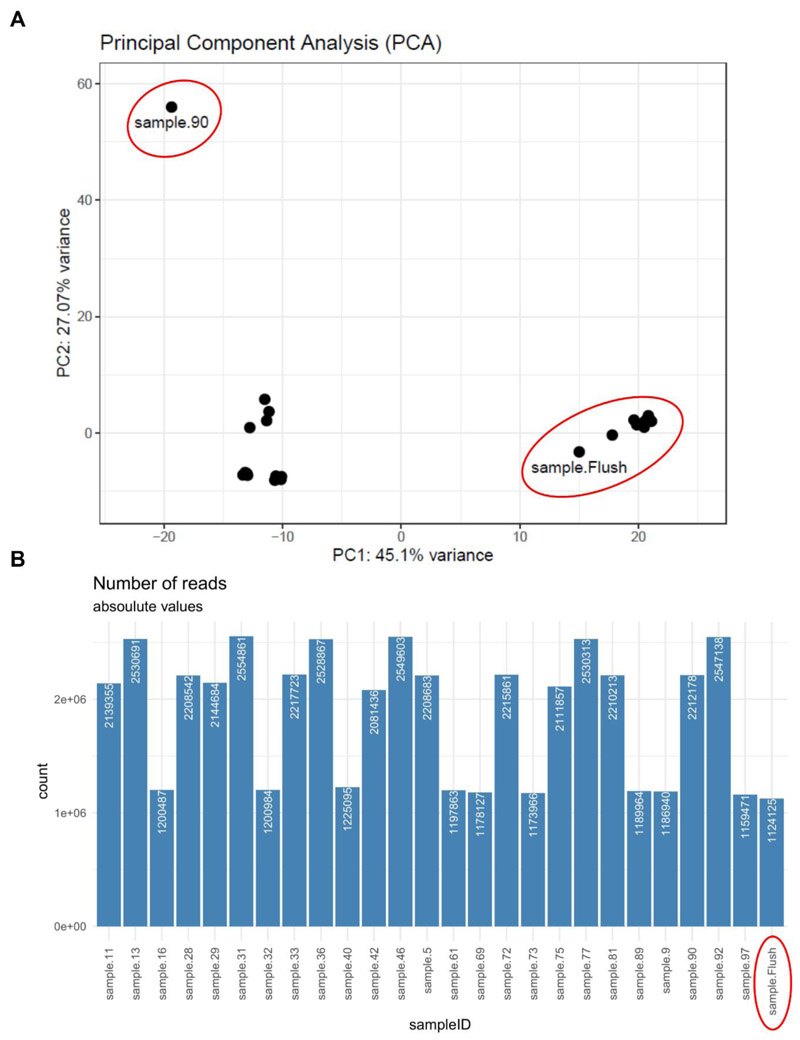
Background profiling. Flushing medium (Flush) for oocyte retrieval was sequenced as background control of FF samples. A) Unsupervised PCA plot of n=25 samples and Flush revealed sample 90 as an outlier and clustering of FF samples with the background control Flush (indicated by red circles). B) Number of raw counts per sample and the medium background control Flush.

Principal component analysis (PCA) revealed a significant deviation from the overall data distribution for sample 90, which was subsequently excluded from further analysis (Figure 1A). Such variations may indicate technical artifacts or biological anomalies, warranting exclusion to ensure the integrity and reliability of downstream analyses. Flushing media (Flush) (ORIGIO, M å l ø v, Denmark), used to aspirate follicles and collect oocytes, was sequenced as a count background control. PCA revealed that the background control Flush clustered with nine FF samples and exhibited comparable miRNA count levels of approximately 1×10^6^ (Figure 1B). In contrast, the other FF samples showed counts twice as high (2×10^6^). To ensure data consistency and focus on biologically relevant samples, only those with higher miRNA counts (2×10^6^) were included in the subsequent analysis and designated as the discovery cohort (n=15).

### Statistical analysis

Background profiling, unsupervised clustering and data normalization was conducted with the statistical program R Version 4.4.1 (19) over the RStudio IDE (20) extended with the package DESeq2 (17). SPSS Statistics-Software V.29 (IBM^®^ SPSS Statistics-Software, NY, USA) and Graph Pad Prism 10.3.0 Software (GraphPad Software Inc., CA, USA) were used for correlations, analysis of variance and graph plotting. Data are presented as box plots displaying the median, minimum, maximum, and the first and third quartiles. All data sets were tested for normal distribution using Shapiro-Wilk and Kolmogorov-Smirnov tests. Depending on the distribution of the data, parametric or non-parametric statistical tests were applied. ANOVA (Bonferroni multiple comparison test) or Kruskall–Wallis test (Dunn’s multiple comparison test) were used to compare the variance between the three clusters. Unpaired t-tests or Mann-Whitney tests were used to compare miRNA counts between groups with different fertility rates.

## Results

### Study participants

After background profiling (Figure 1), miRNA sequencing data from 15 FF samples (discovery cohort) were analyzed. Baseline characteristics, including clinical parameters, body composition and age, are summarized in Table 2. The cohort exhibited a broad range of BMI, FMI, and age, with all participants being metabolically healthy. Notably, ovarian reserve assessments revealed a low AFC, while AMH levels remained within the normal range. The infertility diagnoses for the discovery cohort are detailed in Supplementary Figure 1B.

### Identification of distinct intrafollicular miRNA expression profiles associated with plasma bilirubin levels

**Figure 2:**
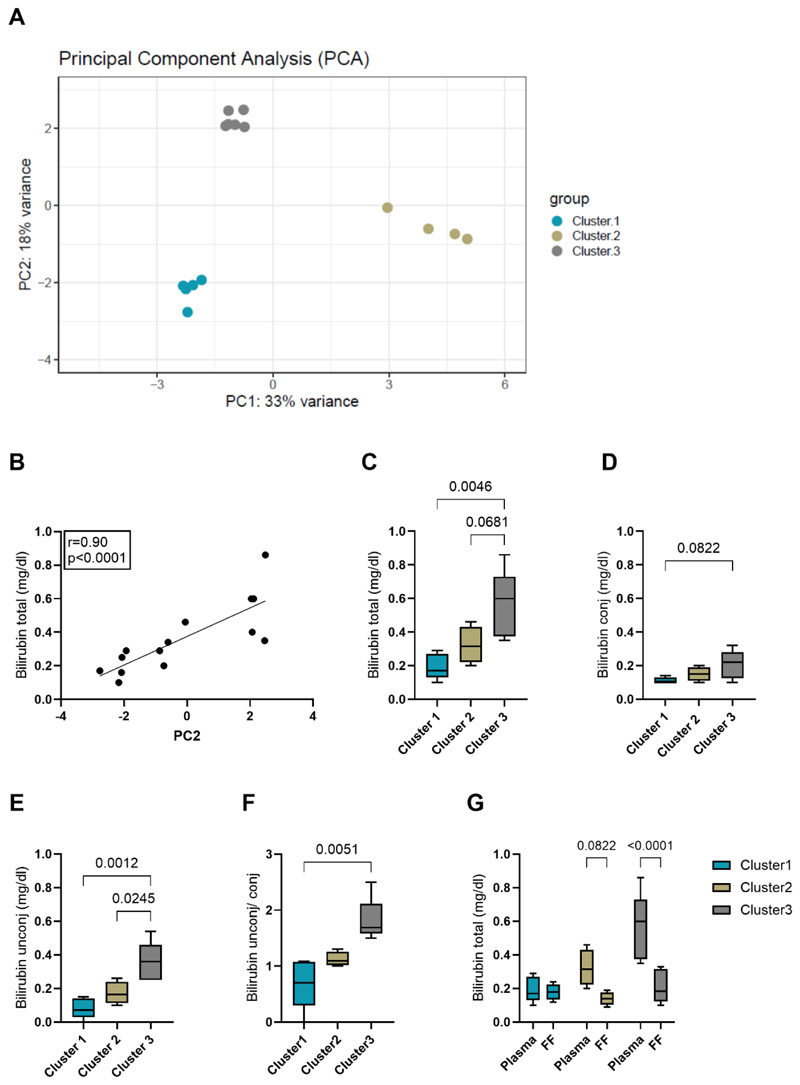
Plasma bilirubin is associated with intrafollicular miRNA cluster separation. miRNA expression in FF samples (n=15) was analyzed by RNA sequencing. A) PCA demonstrated three clusters. B) Plasma levels of total bilirubin correlated significantly with PC2. C-E) Multiple comparison tests of total, conjugated (conj) and unconjugated (unconj) bilirubin plasma levels between clusters were performed using One-way ANOVA and Kruskal-Wallis test, respectively. F) The ratio of unconjugated (unconj) to conjugated (conj) plasma bilirubin levels was calculated (Kruskal-Wallis test).G) Plasma and FF total bilirubin levels were compared (Two-way ANOVA). Data are presented as box-plots showing the median, min, max and quartiles.

PCA revealed three independent miRNA expression clusters (Cluster 1: n=5; Cluster 2: n= 4; Cluster 3: n=6), with PC1 and PC2 accounting for 33% and 18% of the total variance, respectively (Figure 2A). To identify the driver of cluster separation in PC1 and PC2, clinical parameters and body metrics (Table 2) were correlated with PC1 and PC2 coordinates. Although all women had bilirubin in normal range, without cases of hyperbilirubinemia, total bilirubin was the clinical trait that correlated best (highest correlation coefficient) with PC2 (p<0.0001, r = 0.899) (Figure 2B). In contrast, the association between total bilirubin and PC1 was observed as a non-significant trend (p=0.086, r=0.478) (Supplementary Figure 2). To examine differences in plasma bilirubin levels between the three generated clusters, total, conjugated and unconjugated concentrations were studied. Group comparison tests between the clusters revealed that both total as well as unconjugated bilirubin differed substantially between cluster 1 and cluster 3 (total: p=0.005, unconj: p=0.001), respectively (Figure 2C, E). Cluster 3 was also significantly different from cluster 2 for unconjugated bilirubin levels (p=0.024), while total and conjugated levels only showed a trend towards increased levels in cluster 3 compared to the other groups (Figure 2C-E). The ratio of unconjugated to conjugated bilirubin was also calculated as a proxy for functional liver metabolism. Individuals in cluster 3 had the highest unconjugated/conjugated bilirubin ratio and showed a significant difference compared to cluster 1 (p=0.005) (Figure 2F). To address the variability in ovarian stimulation protocols, we have provided a detailed overview of individual regimens in the discovery cohort (Supplementary Table 1). Our analysis revealed no discernible clustering of patients based on similar treatment protocols that could introduce bias into our findings. Given that ovarian stimulation strategies are tailored to individual factors such as ovarian reserve, age, and infertility diagnosis, variability is inherent. To evaluate potential confounders, we analyzed AMH, AFC, and patient age across the three clusters. Additionally, as FSH-induced estrogen elevation may influence bilirubin metabolism, this effect was also assessed. No statistically significant differences were observed for any parameter (Supplementary Figure 3). In addition to plasma bilirubin levels, also intrafollicular bilirubin was measured. Despite the increased plasma bilirubin concentration in cluster 3, total bilirubin in FF remained at a level comparable to the other clusters (Figure 2G). Furthermore, plasma levels of total and unconjugated bilirubin were significantly correlated with total bilirubin levels in FF, respectively (Supplementary Figure 4).

### Plasma bilirubin exhibits a substantial positive correlation with red blood cell indices and a strong inverse relationship with oocyte fertility rates

**Figure 3:**
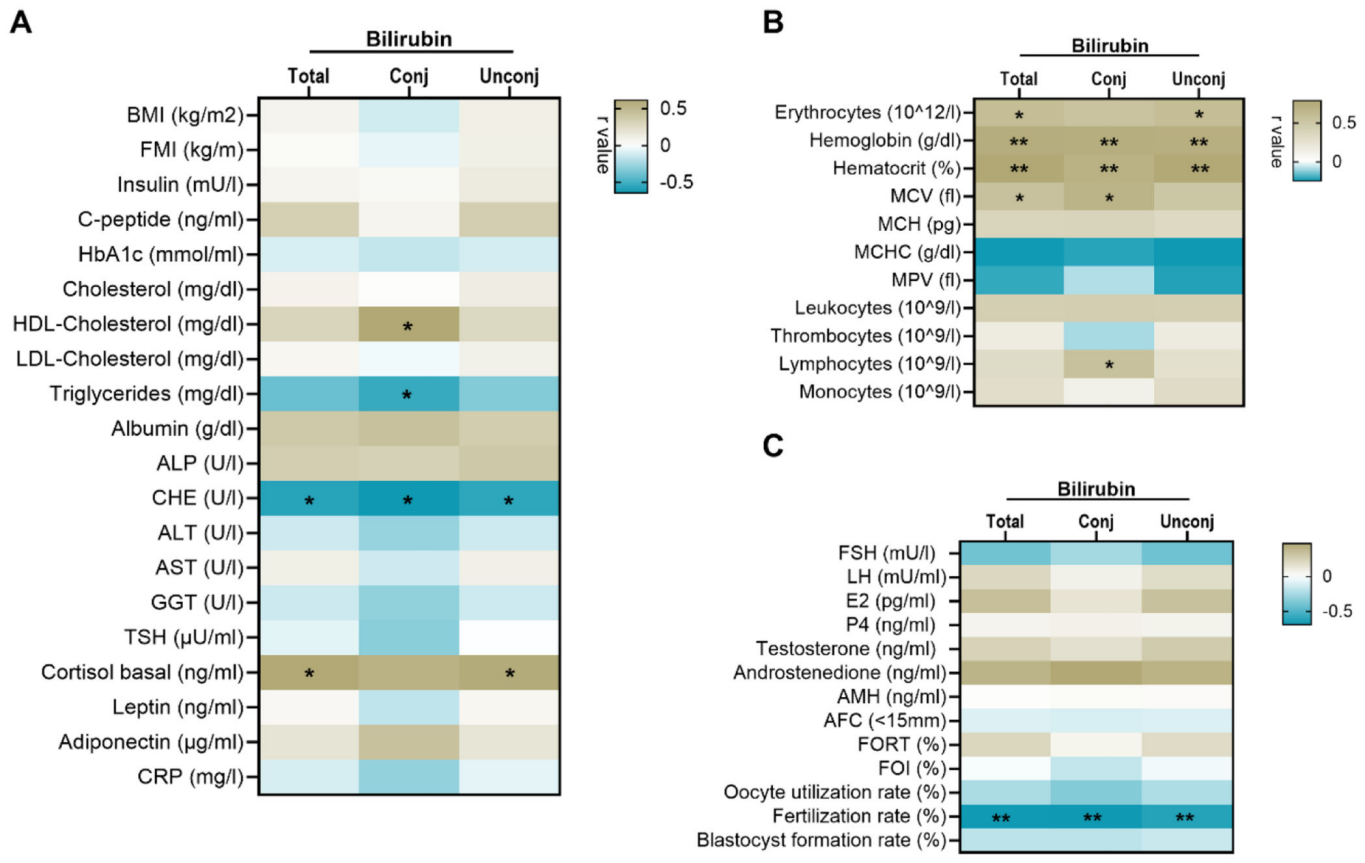
Correlation matrix of bilirubin and representative clinical traits of metabolic (A), blood (B) and IVF (C) parameters. Analysis was performed by Pearson and Spearman correlations, respectively, depending on the normal distribution of data. *p ≤ 0.05; **p ≤ 0.01

We then examined the associations between total, conjugated and unconjugated plasma bilirubin levels and defined clinical parameters. Regarding metabolic parameters, CHE showed an inverse correlation with all forms of bilirubin (total: p=0.028, r=-0.584; conj: p=0.015, r=-0.646; unconj: p=0.036, r=-0.562). Conjugated bilirubin was inversely correlated with triglyceride levels (p=0.048, r=-0.541) and positively associated with HDL-cholesterol (p=0.023, r=0.610). Furthermore, total and unconjugated bilirubin levels were associated with basal cortisol levels in women (total: p=0.023, 0.599; unconj: p=0.028, r=0.583) (Figure 3A). As bilirubin is crucially involved in heme metabolism, we investigated associations between bilirubin and hematological markers. We observed significant positive correlations between total and unconjugated bilirubin and erythrocyte count (p=0.026, r=0.591), as well as direct associations of bilirubin in its total, conjugated and unconjugated forms with hemoglobin (total: p=0.003, r=0.730; conj: p=0.005, r=0.726; unconj: p=0.004, r=0.715) and hematocrit levels (total: p=0.001, r=0.782; conj: p=0.008, r=0.690; unconj: p=0.001, r=0.768) (Figure 3B). Additionally, total and conjugated bilirubin were directly associated with MCV values (total: p=0.041, r=0.549; conj: p=0.011, r=0.668) respectively, and conjugated bilirubin was correlated with lymphocyte count (p=0.044; r=0.550) (Figure 3B). Furthermore, we aimed to explore the potential relationship between plasma bilirubin, hormone levels and fertility outcomes. Importantly, the analysis revealed an inverse relationship between oocyte fertility rates and bilirubin levels (total: p=0.007, r=-0.688; conj: p=0.008, r=-0.691; unconj: p=0.016, r=-0.628), suggesting that elevated plasma bilirubin may influence oocyte development and fertilization potential (Figure 3C).

### Association of plasma bilirubin with intrafollicular miRNAs linked to inflammatory pathways

**Figure 4:**
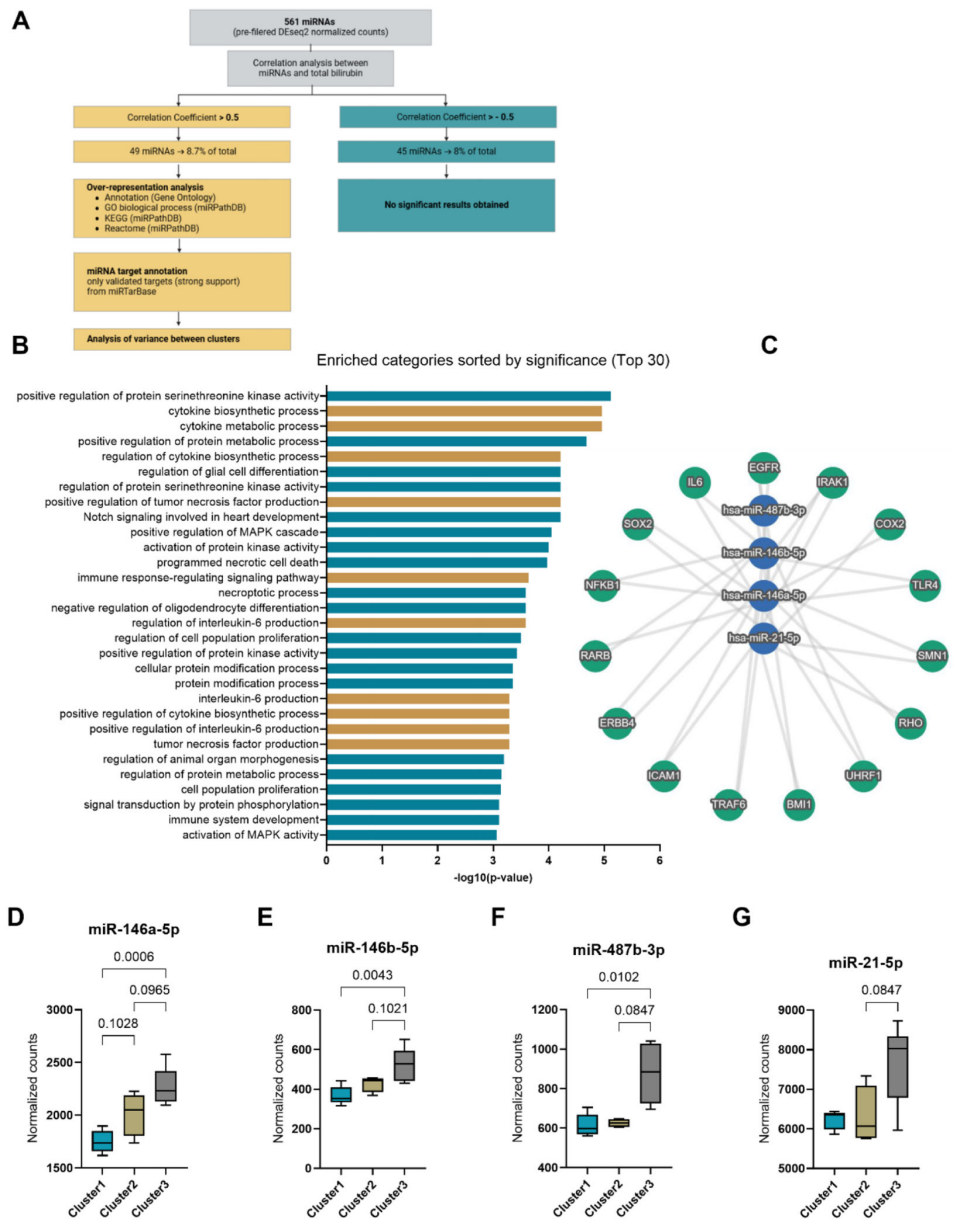
Plasma bilirubin is associated to anti-inflammatory miRNAs in FF. A) Flow chart of data analyses. Correlation analysis was used to pre-select miRNA candidates (Supplementary Table 2 and 3). miEAA enrichment and annotation was performed (miRNAs r>0.5 and r>-0.5) using Over-Representation Analysis (ORA) with following settings: categories of interest were miRNA annotation, GO biological process, KEGG, and Reactome pathway analysis, p-value adjustment method-FDR, significance level 0.05, min two required hits per sub-category. No significant results were obtained of negatively associated miRNAs. B) Significant miRNA enrichment for GO biological processes. Yellow bars indicate cytokine-related terms. Reactome pathway analysis table is disclosed as supplementary table 5. miRNA annotation and KEGG analysis demonstrated no significant results (Supplementary Figure 5). C) miRNA target analysis was performed by applying the filter for only strong evidenced interactions. D)-G) One-way ANOVA or Kruskall–Wallis test using Bonferroni multiple comparison test and Dunn’s multiple comparison test was performed, respectively to analyze the variance of miRNA candidates between the three clusters.

The data analysis workflow to identify significant miRNA pathways is outlined in Figure 4A. Correlation analysis was conducted to evaluate the impact of plasma bilirubin levels on intrafollicular miRNAs. Of 561 pre-filtered mature miRNAs (total read counts of 100 or above across all samples) analyzed, 49 (8.7%) were positively associated with total bilirubin levels, while 45 (8%) were inversely correlated (Supplementary Tables 2 and 3). MicroRNA enrichment and annotation analysis were performed using miEAA (18) platform. Over Representation analysis (ORA) for positively associated miRNAs revealed significant enrichment of GO biological processes encompassing cytokine metabolic and biosynthetic processes, which were identified as top two and three categories (Figure 4B). Furthermore, a total of 10 cytokine related terms were listed in the top 30 significantly enriched processes (Figure 4B). GO biological processes and Reactome pathway analysis revealed key miRNAs enriched in these categories including miR-146a-5p, miR-146b-5p, miR-487b-3p, and miR-21-5p (Supplementary Tables 4 and 5). Target annotation using miRTarBase via the platform miRTarGetLink 2.0 (21) confirmed experimentally validated interactions between these miRNAs and inflammatory markers, including interleukin-6 (IL6), cyclooxygenase 2 (COX2), toll-like receptor 4 (TLR4), interleukin 1 receptor associated kinase 1 (IRAK1) and nuclear factor kappa B (NFKB1) (Figure 4C). A full list of respective targets is presented in supplementary file 2. Analysis of variance between the three clusters revealed that hsa-miR-146a-5p, miR-146b-5p and miR-487b-3p were significantly elevated in cluster 3 compared to cluster 1 (p<0.001; p<0.01; p=0.010), with a trend observed between clusters 2 and 3 (Figure 4D-F). In contrast, miR-21-5p exhibited only a trending difference between clusters 3 and 2 (Figure 4G).

### Inverse relationship between anti-inflammatory miRNAs and oocyte fertility rates

**Figure 5:**
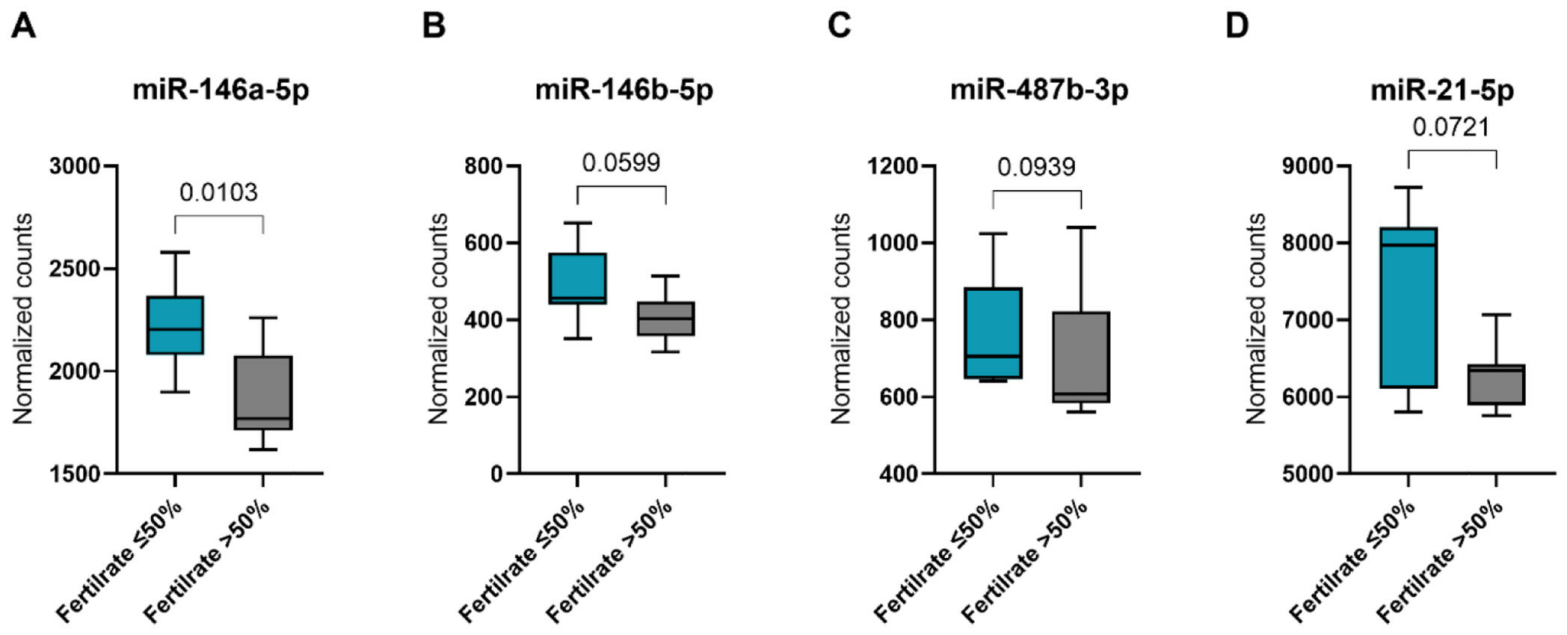
Decreased levels of anti-inflammatory miRNAs in patients with higher fertility rates. The expression levels of (A) miR-146a-5p, (B) miR-146b-5p, (C) miR-487-3p and (D) miR-21-5p were compared among patients with oocyte fertilization rates below, equal to or above 50%. Statistical analyses were performed using unpaired t-tests or Mann-Whitney tests, depending on data distribution. Data are presented as box plots displaying the median, minimum, maximum, and quartiles.

Given the strong negative correlation observed between oocyte fertility rates and plasma bilirubin levels (Figure 3C), we proceeded to investigate the potential impact of anti-inflammatory miRNAs—miR-146a-5p, miR-146b-5p, miR-487b-3p, and miR-21-5p—on fertility rates. The analysis showed a significant decrease in the transcript abundance of miR-146a-5p in FF of patients with a fertility rate >50% (p<0.01) (Figure 5A). Additionally, the transcript levels of miR-146b-5p, miR-487b-3p, and miR-21-5p exhibited a trend towards significant reduction (Figure 5B-D). Association analysis revealed significant inverse correlations for miR-146a-5p and miR-146b-5p with the fertility rate (p<0.01, r=-0.66) (Supplementary Figure 6A, B). Conversely, miR-487b-3p and miR-21-5p exhibited no or a weak inverse correlation with fertility rates (p=0.04, r=-0.54) (Supplementary Figure 6C, D). Additionally, the individual embryo quality per cluster (Supplementary Figure 7), implantation rates for both fresh and cryopreserved single embryo transfers, and live birth rates within one ovarian stimulation cycle were calculated (Supplementary Table 6). Our analysis revealed that Cluster 1 exhibited the poorest reproductive outcomes, with a 0% implantation rate in fresh embryo transfers and only 1 out of 2 cryopreserved transfers resulted in implantation. In contrast, Clusters 2 and 3 demonstrated significantly improved implantation and live birth rates, each achieving a 66.7% success rate. However, the small sample size, the inclusion of both good- and poor-quality embryos from recurrent single transfers within the same patient cycle, and the presence of patients in each cluster who did not develop embryos within their stimulation cycle limit the interpretation of these findings.

## Discussion

Our study shows a novel link between plasma bilirubin levels and miRNAs in FF. Specifically we found that higher levels of total plasma bilirubin were associated with distinct miRNA profiles characterized by an enrichment of anti-inflammatory miRNAs, which target key players of inflammation (e.g. IL6, COX2, TLR4, IRAK1, NFKB1). Moreover, total, conjugated and unconjugated bilirubin levels as well as the set of detected miRNAs were negatively associated with oocyte fertility rates. Therefore, we propose that plasma bilirubin – within physiological range - may play a role in influencing the intrafollicular environment. The potential role of plasma bilirubin in FF and oocyte physiology adds as a novel aspect to the intensively studied role of bilirubin in hemolysis and liver disease.

Bilirubin, long time regarded as merely a byproduct of heme catabolism with limited functionality, has traditionally been seen - when exceeding levels of 1 mg/dl - as an indicator of liver disease or even as a potentially neurotoxic agent (22,23). However, emerging research has established bilirubin as a molecule with potent antioxidant properties and significant metabolic functions even at physiological concentrations (24–29). Recent evidence highlights mildly elevated plasma bilirubin (≥0.588 mg/dl) as a promising biomarker for mitigating cardiometabolic diseases in human and animal models, including obesity and type 2 diabetes mellitus (26,30). While these benefits were initially attributed to bilirubin´s antioxidant properties, recent studies highlight additional mechanisms, such as cell signaling modulation, protein phosphorylation, and activation of nuclear receptors (24,27). Bilirubin also plays an immunomodulatory role, which may contribute to the reduced prevalence of inflammatory, autoimmune, and degenerative diseases observed in individuals with Gilbert’s syndrome (31). Gilbert´s syndrome is characterized by mild unconjugated hyperbilirubinemia, a benign form of hyperbilirubinemia (31). However, the relationship between Gilbert´s syndrome and IVF outcomes has not been systematically investigated in literature. Independent of this condition, bilirubin has been detected in human FF and linked to reproductive outcomes. Interestingly, one study demonstrated a positive correlation between the 455 nm spectrophotometric absorbance in FF — a wavelength associated with yellow pigments such as bilirubin and carotenoids — and oocyte fertilization potential, with bilirubin identified as the primary contributor to this absorbance peak (32). Notably, our data revealed that total, unconjugated and conjugated bilirubin levels were inversely correlated with oocyte fertilization rates. A recent study by Mangione et al. examined the impact of bilirubin levels in FF on clinical parameters of fertilization procedures, comparing FF bilirubin concentrations between fertile and infertile women (33). Consistent with our findings, this study reported a negative correlation between total bilirubin levels and the number of fertilized oocytes, although no association was found between FF and plasma bilirubin levels. Furthermore, it was demonstrated that bilirubin was linked to antioxidant levels and oxidative/nitrosative stress markers in FF (33). Accordingly, the authors hypothesized that increased FF bilirubin levels in infertile females may reflect protective mechanisms against oxidative/nitrosative stress and concomitant elevated inflammation. In our analysis, FF bilirubin concentrations were comparable across clusters, although elevated plasma bilirubin was observed in Cluster 3. This discrepancy may result from a compensatory mechanism that regulates bilirubin transport into the FF, thus maintaining stable levels of this bioactive compound. However, we are aware that such conclusions should be viewed with caution due to the relatively small sample size. In comparison, Mangione et al. analyzed a larger cohort, comprising 35 fertile and 145 infertile women (33). These contradictory findings highlight the need for further investigations to clarify the role of bilirubin in reproductive physiology and its potential impact on fertilization outcomes.

To investigate whether bilirubin is associated with intrafollicular miRNA expression, a correlation analysis of miRNA sequencing data was performed. The analysis revealed that nearly 20% of a total of 561 miRNAs were associated with total plasma bilirubin levels. Pathway enrichment analysis of directly associated miRNAs identified in particular pathways involving cytokine-mediated inflammation. These findings highlight the potential anti-inflammatory role of systemic bilirubin, even within physiological levels. Therefore, we focused our further analysis on four specific miRNAs that were enriched in 10 of the top 30 significantly enriched inflammation-related pathways. Among these, miR-146a-5p and miR-21-5p are well characterized as inflammamiRs, i.e. miRNAs that function as key anti-inflammatory regulators (34). miR-146a-5p plays a pivotal role in modulating key inflammatory pathways, primarily through the regulation of NFKB, IRAK1, and tumor necrosis factor receptor-associated factor 6 (TRAF6) (35). Similarly, miR-21-5p functions as a critical negative regulator of IRAK1 and myeloid differentiation primary response 88 (MYD88) within the TLR signaling pathway (36). Importantly, PPARα has been identified as direct target of miR-21-5p repression, whereas bilirubin serves as an activator of this signaling axis (28,37). This may suggest that miR-21-5p serves as a regulatory feedback mechanism to attenuate bilirubin-mediated PPARα activation. Interestingly, miR-21-5p was the only miRNA among the four analyzed miRNAs enriched in inflammation-related pathways that did not differ within the three generated clusters. However, a trend for distinct miR-21-5p levels was observed between clusters 2 and 3. Although miR-146b-5p and miR-487b-3p are not traditionally classified as inflammamiRs, a growing body of evidence highlights their anti-inflammatory potential (38,39). Notably, miR-146a-5p was significantly decreased in patients characterized by a fertility rate of >50% and demonstrated, together with miR-146b-5p, a strong inverse correlation with oocyte fertilization rates, whereas miR-21-5p showed only a modest association. Thus, a role for miR-146a-5p and miR-146b-5p in modulating oocyte maturation and developmental competence by altering intrafollicular inflammatory processes may be hypothesized. The negative association of these anti-inflammatory miRNAs as well as of bilirubin with fertilization rate may be due to the fact that a subtle physiological inflammation is essential for folliculogenesis and ovulation, involving a precisely orchestrated repertoire of cytokines and eicosanoids (40).

Research into the role of bilirubin in cardiovascular protection has shown that elevated bilirubin levels are associated with favourable changes in lipid profiles, contributing to reduced cardiovascular risk. For instance, patients with Gilbert’s syndrome exhibit significantly lower levels of pro-atherogenic markers, including LDL, triacylglycerol, and total cholesterol (31). Our findings are in line with previous studies, demonstrating a negative association between conjugated bilirubin and triglyceride levels, while conjugated bilirubin was positively correlated with HDL-cholesterol. Furthermore, we observed an indirect association between total, conjugated, and unconjugated bilirubin concentrations and CHE activity. Beyond its traditional role as a marker for liver function, altered CHE activity has been implicated in the pathophysiology of inflammation, and may serve as an early biomarker of inflammatory processes (41). The observed associations suggest a potential link between liver function, systemic inflammation, and the known anti-inflammatory and antioxidant properties of bilirubin.

An important finding of this study was the detection of a substantial number of miRNAs in the flushing medium used during oocyte retrieval and washing procedures. These miRNAs established a remarkable baseline across all samples, and the selection of samples with miRNA levels above this baseline was critical for subsequent analyses. Beyond interfering analysis, miRNAs as regulatory molecules present in flushing medium may potentially influence IVF parameters. This observation underscores a potentially significant yet under-explored avenue of research, focusing on the presence and regulatory roles of miRNAs in routine IVF equipment and their impact on fertilization competence.

A major limitation of our study is the relatively small sample size and the uneven distribution of cluster sizes, which may limit the generalizability of the findings. Furthermore, all study participants underwent ovarian stimulation, receiving an individual stimulation protocol which could introduce a bias in results that may significantly differ from the natural cycle. Nevertheless, as an exploratory analysis, our results provide a valuable basis for future investigations, including larger-scale sequencing studies and *in vitro* experiments, to confirm and extend our observations. Our findings underscore the complex interplay between bilirubin and miRNA-mediated regulatory networks and provide new insights into their potential roles in modulating inflammation and oocyte health. Future research should aim to validate these findings in larger cohorts and assess the functional roles of the identified miRNAs in oocyte biology.

## Abbreviations

AFC: Antral follicle count
ALT: Alanine aminotransferase
ALP: Alkaline phosphatase
AMH: Anti-Müllerian hormone
AST: Aspartate aminotransferase
ART: Assisted reproductive technologies
CHE: Cholinesterase
Conj: Conjugated
CRP: C-reactive protein
COX2: Cyclooxygenase 2
E2: Estradiol
Flush: Flushing media
FOI: Follicle to oocyte index
FF: Follicular fluid
FORT: Follicular Output Rate
FSH: Follicle stimulating hormone
GGT: Gamma-glutamyltransferase
HbA1c: Glycated hemoglobin
HDL-cholesterol: High-density lipoprotein-cholesterol
IVF: *in vitro* fertilization
IL6: Interleukin-6
IRAK1: Interleukin 1 Receptor Associated Kinase 1
LDL-cholesterol: Low-density-lipoprotein-cholesterol
LH: Luteinizing hormone
MCHC: Mean corpuscular hemoglobin concentration
MCH: Mean corpuscular hemoglobin
MCV: Mean individual erythrocyte volume
MPV: Mean platelet volume
miRNA: MicroRNA
NFKB1: Nuclear factor kappa beta
P4: Progesterone
PCOS: Polycystic ovary syndrome
PCA: Principal component analysis
TSH: Thyroid-stimulating hormone
TLR4: Toll-like receptor 4
Unconj: Unconjugated

## Supplementary Material

Supplementary Figures

Supplementary Tables

## Figures and Tables

**Table 1:** Subject characteristics of the pilot cohort.

Pilot cohort (n=25)		
	Parameters	Median, IQR
Anthropometric and demographic parameters	Age (y)	35 (32-37)
	BMI (kg/m^2^)	24.4 (21.7-27.4)
	FMI (kg/m)	9 (6.3-11.9)
Glucometabolic parameters	Insulin (mU/l)	8.4 (5.9-11.9)
	C-peptide (ng/ml)	1.7 (1.4-2.2)
	HbA1c (mmol/ml)	33 (32-35)
Lipid metabolism	Cholesterol (mg/dl)	179 (156.3-197.8)
	HDL-Cholesterol (mg/dl)	63.5 (52.5-74.5)
	LDL-Cholesterol (mg/dl)	97 (75-115.5)
	Triglycerides (mg/dl)	93.5 (72-111.8)
Liver function	Bilirubin total (mg/dl)	0.34 (0.29-0.50)
	Bilirubin conjugated (mg/dl)	0.15 (0.1-0.2)
	Bilirubin unconjugated (mg/dl)	0.18 (0.13-0.33)
	Bilirubin unconjugated/conjugated	1.18 (0.95-1.68)
	Albumin (g/dl)	4.4 (4.1-4.8)
	ALP (U/l)	53.3 (47.2-60.5)
	CHE (U/l)	6692 (5692-7899)
	ALT (U/l)	17 (14-34.2)
	AST (U/l)	20.5 (17.2-31.7)
	GGT (U/l)	13 (9.2-17-7)
Hormones, adipokines and inflammatory markers	TSH (μU/ml)	2.2 (1.6-3.1)
	Cortisol (ng/ml)	158.1 (123.7-196.8)
	Leptin (ng/ml)	12.3 (6.7-23.5)
	Adiponectin (μg/ml)	9.2 (7.5-10.5)
	CRP (mg/l)	1.7 (1.3-2.4)
Ovarian reserve parameters	AFC (<15 mm)	4 (3.0-7.7)
	AMH (ng/ml)	2.3 (0.8-3.3)

Values are reported as median and interquartile range (IQR). Abbreviations: BMI, body mass index; FMI, fat mass index; HbA1c, glycated hemoglobin; HDL-cholesterol, high-density lipoprotein-cholesterol; LDL-cholesterol, low-density-lipoprotein-cholesterol; ALP, alkaline phosphatase; CHE, cholinesterase; ALT, alanine aminotransferase; AST, aspartate aminotransferase; GGT, gamma-glutamyltransferase; TSH, thyroid-stimulating hormone, CRP, C-reactive protein; AFC, antral follicle count; AMH, Anti-mullerian hormone.

**Table 2:** Subject baseline characteristics of the discovery cohort used for analysis.

Discovery cohort (n=15)		
	Parameters	Median, IQR
Anthropometric and demographic parameters	Age (y)	34 (31–37)
	BMI (kg/m2)	25.8 (23.2-29.7)
	FMI (kg/m)	9.5 (8.0- 13.8)
Glucometabolic parameters	Insulin (mU/l)	11.0 (7.5-13.1)
	C-peptide (ng/ml)	1.9 (1.5-2.5)
	HbA1c (mmol/ml)	33(32-35)
Lipid metabolism	Cholesterol (mg/dl)	184 (153.8-204.3)
	HDL-Cholesterol (mg/dl)	61 (51.7-70.5)
	LDL-Cholesterol (mg/dl)	104.5 (73.2-119.3)
	Triglycerides (mg/dl)	93.5 (72-111.3)
Liver function	Bilirubin total (mg/dl)	0.31 (0.19-0.50)
	Bilirubin conjugated (mg/dl)	0.14 (0.1-0.2)
	Bilirubin unconjugated (mg/dl)	0.16 (0.09-0.28)
	Bilirubin unconjugated/conjugated	1.1 (0.92-1.67)
	Albumin (g/dl)	42.5 (40-45.7)
	ALP (U/l)	57.5 (48.5-68)
	CHE (U/l)	6692 (6080-7972)
	ALT (U/l)	16 (13-32.7)
	AST (U/l)	19.5 (15.7-26.5)
	GGT (U/l)	13 (9.7-17.2)
Hormones, adipokines and inflammatory markers	TSH (μU/ml)	2.1 (1.7-2.6)
	Cortisol (ng/ml)	148.3 (127.7-176.7)
	Leptin (ng/ml)	21.3 (11.5-27.9)
	Adiponectin (μg/ml)	9.35 (7.7-10.7)
	CRP (mg/l)	2.1 (1.7-7.6)
Ovarian reserve parameters	AFC (<15 mm)	4 (2.5-8.2)
	AMH (ng/ml)	1.7 (0.5-3.3)

Values are reported as median and interquartile range (IQR). Abbreviations: BMI, body mass index; FMI, fat mass index; HbA1c, glycated hemoglobin; HDL-cholesterol, high-density lipoprotein-cholesterol; LDL-cholesterol, low-density-lipoprotein-cholesterol; ALP, alkaline phosphatase; CHE, cholinesterase; ALT, alanine aminotransferase; AST, aspartate aminotransferase; GGT, gamma-glutamyltransferase; TSH, thyroid-stimulating hormone, CRP, C-reactive protein; AFC, antral follicle count; AMH, Anti-mullerian hormone.

## Data Availability

Data from this study were deposited at the National Center for Biotechnology Information Sequence Read Archive under the accession number PRJNA1188531.

## Box 1 New & Noteworthy

Maternal plasma bilirubin levels are associated with a distinct anti-inflammatory miRNA signature in follicular fluid that targets key inflammatory mediators. This signature is diminished in patients with higher fertilization rates, suggesting a novel role for plasma bilirubin in potentially modulating the ovarian follicular environment.
