# Supplementary material for: Novel link between plasma bilirubin and anti-inflammatory miRNA profiles in follicular fluid of IVF patients
Source: Am J Physiol Endocrinol Metab. Author manuscript; Available in PMC 2025 Jun 9. (PMC7617750; doi:10.1152/ajpendo.00479.2024)
Supplement: Supplementary Figures [file EMS205252-supplement-Supplementary_Figures.pdf]

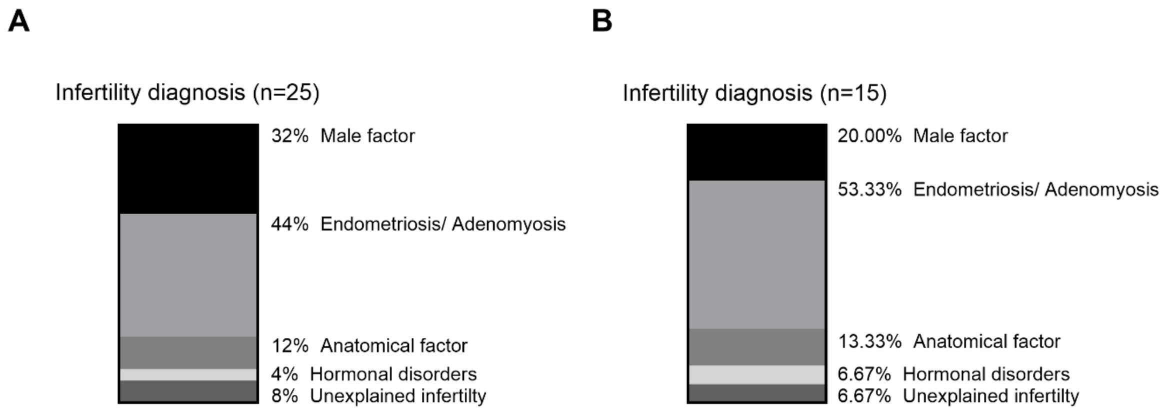

Supplementary Figure 1. Distribution of infertility diagnoses. Diagnosis of infertility is expressed as % of a total of n=25 patients (A, pilot cohort) and n=15 patients (B, discovery cohort).

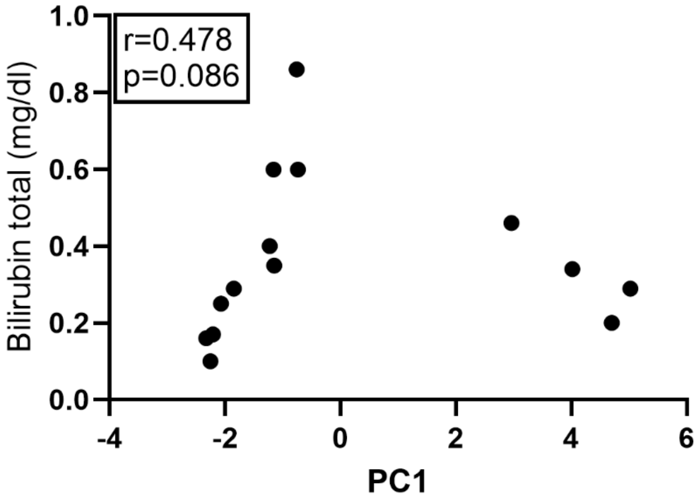

Supplementary Figure 2. Association between bilirubin and PC1. Spearman correlation between PC1 (33% variance) and total bilirubin levels in plasma.

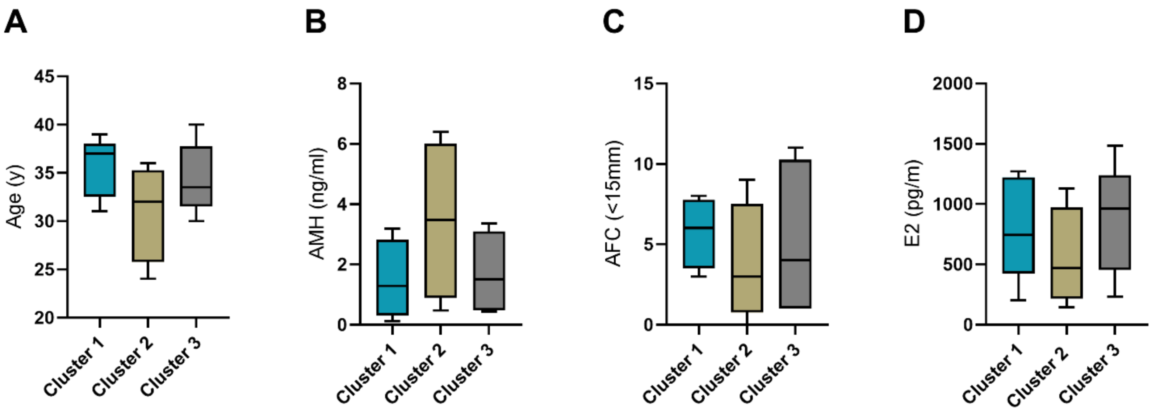

Supplementary Figure 3. Impact of confounding factors on cluster separation. Ovarian reserve parameters (AMH, AFC), age of the patients and serum estradiol levels between the clusters were analyzed by One-way ANOVA.

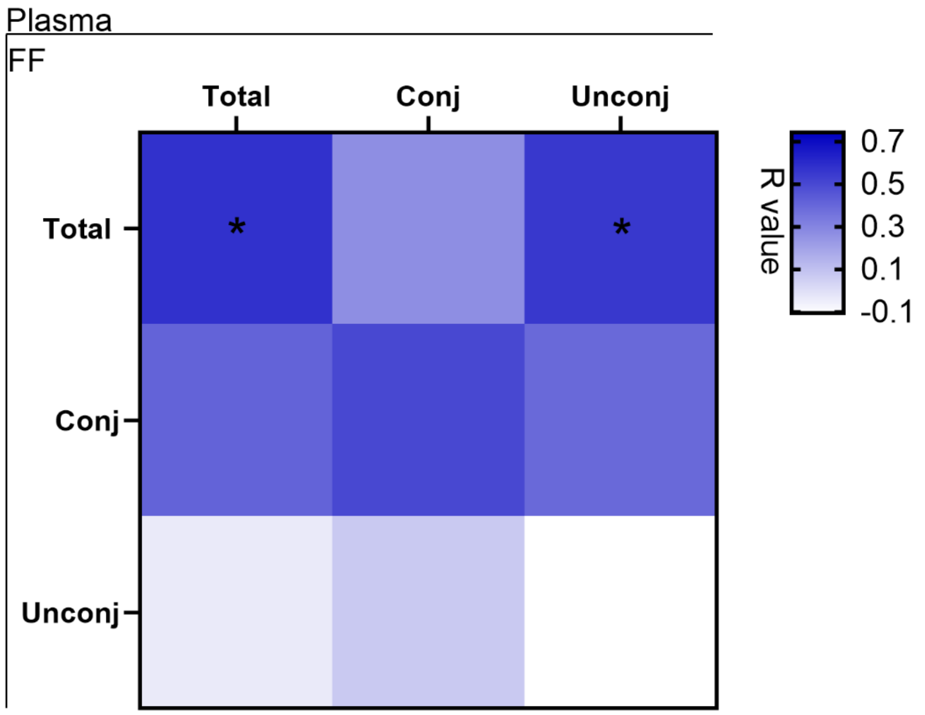

Supplementary Figure 4. Correlation matrix of plasma and FF total, conjugated (conj) and unconjugated (unconj) bilirubin levels. Pearson and Spearman correlations were performed to determine associations between plasma and FF bilirubin levels, respectively.

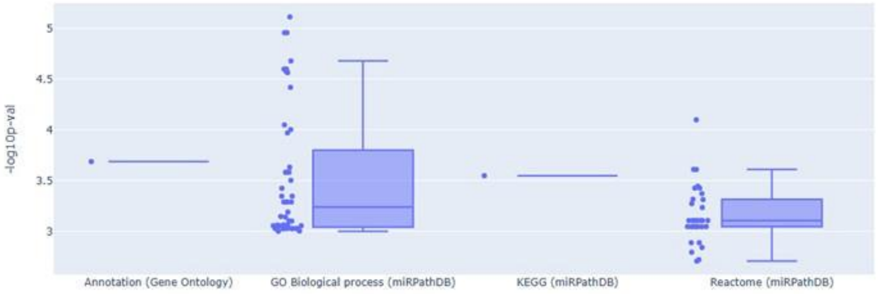

Supplementary Figure 5. GO biological process and Reactome pathway analysis revealed significantly enriched categories. Overrepresentation analysis using bilirubin associated miRNAs (r>0.5) was performed by applying annotation, GO biological processes, KEGG and Reactome pathway analysis categories. The analysis resulted in significant enriched pathways for GO biological processes and Reactome pathways, compared to annotation and KEGG categories.

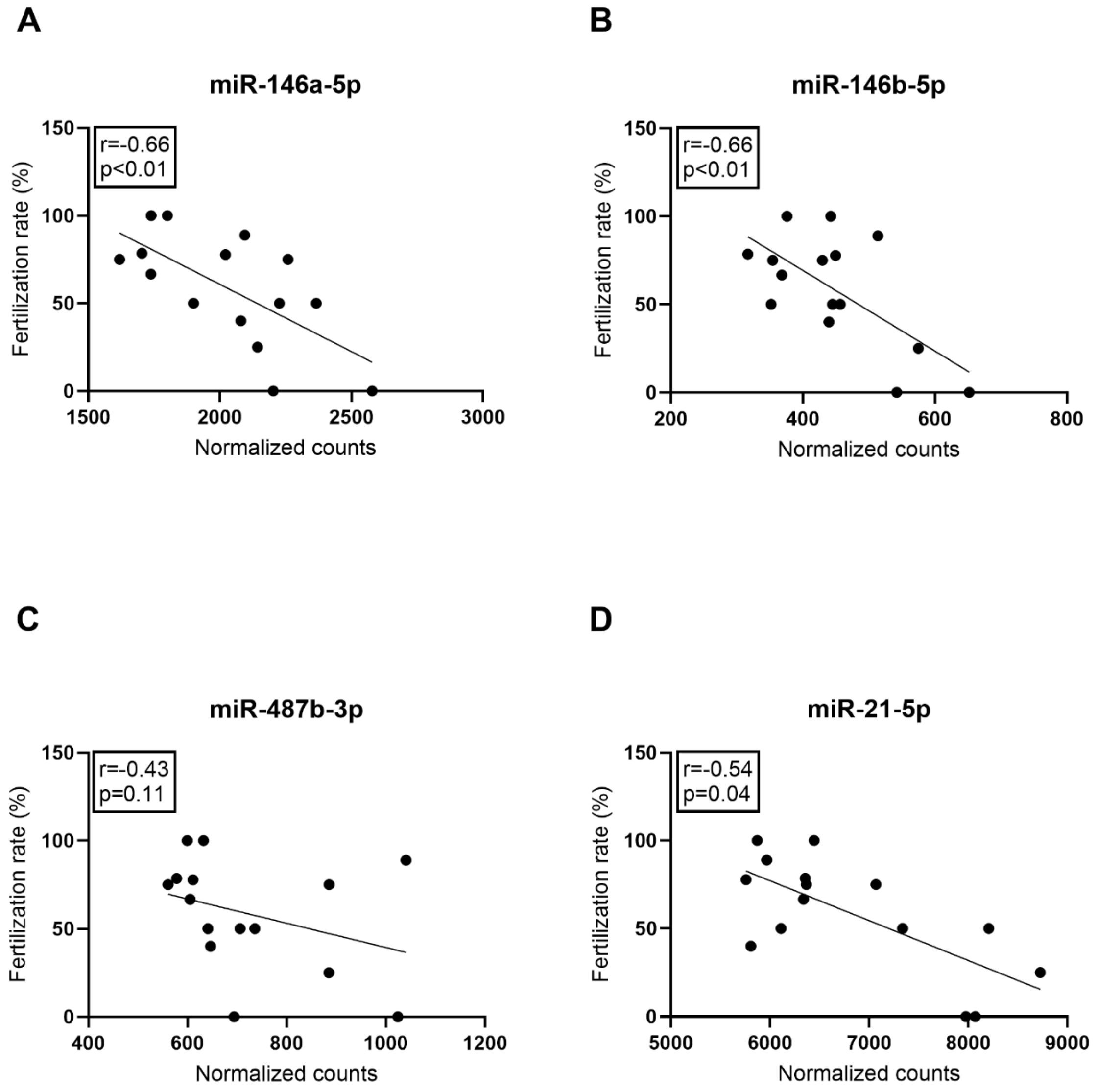

Supplementary Figure 6. Anti-inflammatory miRNAs are inversely associated with the fertility rates of oocytes. Fertility rate was defined as the number of oocytes with two pronuclei divided by the number of all oocytes collected. Pearson and Spearman correlation analysis was applied to investigate the link between miRNA candidates and fertility rates.

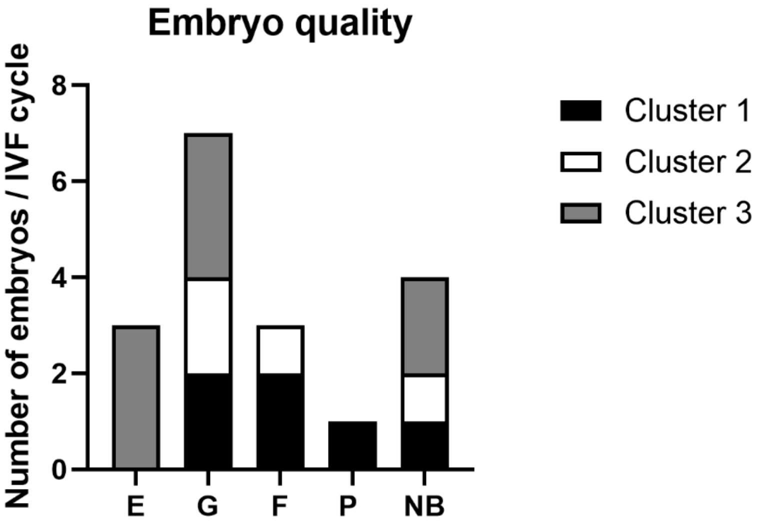

Supplementary Figure 7. Presentation of embryo qualities per cluster. Excellent (E); good (G); fair (F); poor (P); non blast (NB);
