# Supplementary material for: Novel link between plasma bilirubin and anti-inflammatory miRNA profiles in follicular fluid of IVF patients
Source: Am J Physiol Endocrinol Metab. Author manuscript; Available in PMC 2025 Jun 9. (PMC7617750; doi:10.1152/ajpendo.00479.2024)
Supplement: Supplementary Tables [file EMS205252-supplement-Supplementary_Tables.pdf]

| | | |
|---|---|---|
| | CRP (mg/l) | 2.1 (1.7-7.6) |
| Ovarian reserve parameters | AFC (<15 mm) | 4 (2.5-8.2) |
| | AMH (ng/ml) | 1.7 (0.5-3.3) |

Values are reported as median and interquartile range (IQR). Abbreviations: BMI, body mass index; FMI, fat mass index; HbA1c, glycated hemoglobin; HDL-cholesterol, high-density lipoprotein-cholesterol; LDL-cholesterol, low-density-lipoprotein-cholesterol; ALP, alkaline phosphatase; CHE, cholinesterase; ALT, alanine aminotransferase; AST, aspartate aminotransferase; GGT, gamma-glutamyltransferase; TSH, thyroid-stimulating hormone, CRP, C-reactive protein; AFC, antral follicle count; AMH, Anti-mullerian hormone.

**Supplementary Table 1.** Overview of patient characteristics and ovarian stimulation protocols of the discovery cohort (n=15).

| Cluster | Sample | Infertility diagnosis | Stimulation (days) | Ovarian stimulation medications | Trigger shots |
|---|---|---|---|---|---|
| **1** | 5 | Male factor | 9 | Ovaleap 150 IE | Decapetyl 0.1 mg |
| | 28 | Tubal factor | 9 | Menopur 300 IE, Cetrotide 0.25 mg | Zivafert 10.000 IE |
| | 33 | Endometriosis/ Adenomyosis | 13 | Meriofert 300 IE, Decapeptyl 0.05 mg | Zivafert 10.000 IE |
| | 72 | Endometriosis /Adenomyosis | 13 | Meriofert 300 IE, Decapeptyl 0.05 mg | Zivafert 10.000 IE |
| | 81 | Tubal factor | 10 | Gonal F 250 IE, Cetrotide 0.25 mg | Zivafert 10.000 IE |
| **2** | 11 | Endometriosis /Adenomyosis | 11 | Meriofert 300 IE, Decapeptyl 0.1 mg | Zivafert 10.000 IE |
| | 29 | Endometriosis /Adenomyosis | 13 | Gonal F 150IE, Cetrotide 0.25 mg | Zivafert 10.000 IE |
| | 46 | Endometriosis /Adenomyosis | 11 | Gonal F 300 IE, Meriofert 300 IE, Ganirelix 0.25 mg | Zivafert 10.000 IE |
| | 75 | Male factor | 11 | Rekovelle 12 mg, Orgalutran 0.25 mg | Zivafert 10.000 IE |
| **3** | 13 | Endometriosis /Adenomyosis | 11 | Menopur 300 IE, Cetrotide 0.25 mg | Zivafert 10.000 IE |
| | 31 | Unexplained infertility | 8 | Meriofert 150 IE, Cetrotide 0.25 mg | Zivafert 10.000 IE |
| | 36 | Endometriosis /Adenomyosis | 10 | Meriofert 225 IE, Ganirelix 0.25 mg | Decapetyl 0.1 mg |
| | 42 | Hypogonadotropic hypogonadism | 12 | Menopur 100 IE, Cetrotide 0.25 mg | Decapeptyl 0.1 mg, Zivafert 5.000 IE |
| | 77 | Endometriosis /Adenomyosis | 12 | Gonal F 225 IE, Cetrotide 0.25 mg | Zivafert 10.000 IE |
| | 92 | Male factor | 11 | Rekovelle 12 mg, Cetrotide 0.25 mg | Zivafert 10.000 IE |

**UPDATED August 2024**

**Supplementary Table 2.** List of miRNAs that were positively associated with total bilirubin levels in plasma. Normalized miRNA counts were tested for normal distribution using Shapiro-Wilk and Kolmogorov-Smirnov tests. Depending on the distribution of the data, either Pearson correlation or Spearman correlation analysis was applied. miRNAs exhibiting a correlation coefficient r> 0.5 were included for further analysis.

| **r> 0.5** | **r** | **p value** |
|---|---|---|
| hsa-miR-1537-3p | 0.631 | 0.015 |
| hsa-miR-3617-5p | 0.615 | 0.019 |
| hsa-miR-28-5p | 0.893 | 0.000 |
| hsa-miR-940 | 0.660 | 0.010 |
| hsa-miR-410-3p | 0.717 | 0.004 |
| hsa-miR-656-3p | 0.564 | 0.036 |
| hsa-miR-362-5p | 0.591 | 0.026 |
| hsa-miR-487b-3p | 0.599 | 0.024 |
| hsa-miR-889-5p | 0.711 | 0.004 |
| hsa-miR-4286 | 0.727 | 0.003 |
| hsa-miR-544b | 0.662 | 0.010 |
| hsa-miR-766-3p | 0.965 | 0.000 |
| hsa-miR-136-5p | 0.750 | 0.002 |
| hsa-miR-17-3p | 0.688 | 0.007 |
| hsa-miR-20a-3p | 0.676 | 0.008 |
| hsa-miR-409-5p | 0.626 | 0.017 |
| hsa-miR-9-3p | 0.537 | 0.047 |
| hsa-miR-376b-3p | 0.729 | 0.003 |
| hsa-miR-4433b-5p | 0.602 | 0.023 |
| hsa-miR-576-5p | 0.661 | 0.010 |
| hsa-miR-28-3p | 0.594 | 0.025 |
| hsa-miR-301a-3p | 0.559 | 0.038 |
| hsa-miR-1273h-3p | 0.634 | 0.015 |
| hsa-miR-4665-5p | 0.707 | 0.005 |
| hsa-miR-12136 | 0.540 | 0.046 |
| hsa-miR-382-5p | 0.581 | 0.029 |
| hsa-miR-3074-3p | 0.585 | 0.028 |
| hsa-miR-4710 | 0.634 | 0.015 |
| hsa-miR-18b-5p | 0.595 | 0.025 |
| hsa-miR-6882-5p | 0.555 | 0.039 |
| hsa-miR-101-3p | 0.663 | 0.010 |
| hsa-miR-15a-5p | 0.720 | 0.004 |
| hsa-miR-21-5p | 0.670 | 0.009 |
| hsa-miR-6514-5p | 0.555 | 0.040 |
| hsa-miR-654-3p | 0.565 | 0.035 |

**UPDATED August 2024**

| | | |
|---|---|---|
| hsa-miR-128-3p | 0.657 | 0.011 |
| hsa-miR-338-3p | 0.557 | 0.038 |
| hsa-miR-1285-3p | 0.615 | 0.019 |
| hsa-miR-493-3p | 0.733 | 0.003 |
| hsa-miR-146b-5p | 0.732 | 0.003 |
| hsa-miR-146a-5p | 0.802 | 0.001 |
| hsa-miR-769-5p | 0.584 | 0.028 |
| hsa-miR-24-3p | 0.551 | 0.041 |
| hsa-miR-6842-5p | 0.535 | 0.049 |
| hsa-miR-30c-5p | 0.554 | 0.040 |
| hsa-miR-142-3p | 0.655 | 0.011 |
| hsa-miR-27b-3p | 0.617 | 0.019 |
| hsa-miR-4443 | 0.590 | 0.026 |
| hsa-miR-6516-5p | 0.643 | 0.013 |

**Supplementary Table 3.** List of miRNAs that were negatively associated with total bilirubin levels in plasma. Normalized miRNA counts were tested for normal distribution using Shapiro-Wilk and Kolmogorov-Smirnov tests. Depending on the distribution of the data, either Pearson correlation or Spearman correlation analysis was applied. miRNAs exhibiting a correlation coefficient r> -0.5 were included for further analysis.

| **r> -0.5** | **r** | **p value** |
|---|---|---|
| hsa-miR-320d | -0.670 | 0.009 |
| hsa-miR-320b | -0.632 | 0.015 |
| hsa-miR-548l | -0.681 | 0.007 |
| hsa-miR-522-3p | -0.696 | 0.006 |
| hsa-miR-518e-3p | -0.535 | 0.049 |
| hsa-miR-452-5p | -0.639 | 0.014 |
| hsa-miR-512-3p | -0.678 | 0.008 |
| hsa-miR-520f-3p | -0.822 | 0.000 |
| hsa-miR-501-3p | -0.733 | 0.003 |
| hsa-miR-520h | -0.601 | 0.023 |
| hsa-miR-520g-3p | -0.651 | 0.012 |
| hsa-miR-3179 | -0.663 | 0.010 |
| hsa-miR-655-5p | -0.796 | 0.001 |
| hsa-miR-31-5p | -0.586 | 0.028 |
| hsa-miR-410-5p | -0.548 | 0.043 |
| hsa-miR-3143 | -0.612 | 0.020 |
| hsa-miR-4766-3p | -0.610 | 0.020 |
| hsa-miR-1185-2-3p | -0.592 | 0.026 |
| hsa-miR-424-3p | -0.645 | 0.013 |
| hsa-miR-519d-3p | -0.568 | 0.034 |

**UPDATED August 2024**

| | | |
|---|---|---|
| hsa-miR-941 | -0.537 | 0.047 |
| hsa-miR-3154 | -0.559 | 0.038 |
| hsa-miR-16-2-3p | -0.612 | 0.020 |
| hsa-miR-193b-5p | -0.595 | 0.025 |
| hsa-miR-6802-5p | -0.548 | 0.042 |
| hsa-miR-30a-3p | -0.758 | 0.002 |
| hsa-miR-129-5p | -0.590 | 0.026 |
| hsa-miR-144-5p | -0.564 | 0.036 |
| hsa-miR-4516 | -0.819 | 0.000 |
| hsa-miR-1224-5p | -0.652 | 0.012 |
| hsa-miR-132-3p | -0.667 | 0.009 |
| hsa-miR-7851-3p | -0.879 | 0.000 |
| hsa-miR-195-5p | -0.581 | 0.029 |
| hsa-miR-183-5p | -0.700 | 0.005 |
| hsa-miR-1323 | -0.663 | 0.010 |
| hsa-miR-6513-3p | -0.698 | 0.005 |
| hsa-miR-6764-5p | -0.632 | 0.015 |
| hsa-miR-340-3p | -0.568 | 0.034 |
| hsa-miR-4738-3p | -0.670 | 0.009 |
| hsa-let-7c-5p | -0.618 | 0.018 |
| hsa-miR-4533 | -0.738 | 0.003 |
| hsa-miR-1290 | -0.817 | 0.000 |
| hsa-miR-193a-5p | -0.700 | 0.005 |
| hsa-miR-3190-3p | -0.558 | 0.038 |
| hsa-miR-450b-5p | -0.714 | 0.004 |

**Supplementary Table 4.** GO Biological processes analysis revealed significant enrichment of inflammatory processes. Top 10 over-represented GO Biological processes. ↑ over-represented

| GO Biological process (miRPathDB) | Enrichment | P-value | P-adjusted | miRNAs/precursors |
|---|---|---|---|---|
| cytokine biosynthetic process | ↑ | 1.10e-5 | 0.008 | hsa-miR-487b-3p; hsa-miR-21-5p; hsa-miR-146b-5p; hsa-miR-146a-5p |
| cytokine metabolic process | ↑ | 1.10e-5 | 0.008 | hsa-miR-487b-3p; hsa-miR-21-5p; hsa-miR-146b-5p; hsa-miR-146a-5p |
| positive regulation of protein metabolic process | ↑ | 2.09e-5 | 0.008 | hsa-miR-3617-5p; hsa-miR-487b-3p; hsa-miR-301a-3p; hsa-miR-101-3p; hsa-miR-15a-5p; hsa-miR-21-5p; hsa-miR-128-3p; hsa-miR-146a-5p; hsa-miR-24-3p; hsa-miR-30c-5p; hsa-miR-142-3p; hsa-miR-27b-3p |

**UPDATED August 2024**

| | | | | |
|---|---|---|---|---|
| positive regulation of protein serinethreonine kinase activity | ↑ | 7.71e-6 | 0.008 | hsa-miR-410-3p; hsa-miR-487b-3p; hsa-miR-9-3p; hsa-miR-101-3p; hsa-miR-21-5p; hsa-miR-128-3p; hsa-miR-493-3p; hsa-miR-146a-5p; hsa-miR-24-3p; hsa-miR-27b-3p |
| positive regulation of tumor necrosis factor production | ↑ | 2.73e-5 | 0.008 | hsa-miR-487b-3p; hsa-miR-21-5p; hsa-miR-146a-5p |
| regulation of cytokine biosynthetic process | ↑ | 2.52e-5 | 0.008 | hsa-miR-487b-3p; hsa-miR-21-5p; hsa-miR-146b-5p; hsa-miR-146a-5p |
| regulation of glial cell differentiation | ↑ | 2.52e-5 | 0.008 | hsa-miR-3617-5p; hsa-miR-410-3p; hsa-miR-9-3p; hsa-miR-146a-5p |
| regulation of protein serinethreonine kinase activity | ↑ | 2.66e-5 | 0.008 | hsa-miR-410-3p; hsa-miR-17-3p; hsa-miR-9-3p; hsa-miR-101-3p; hsa-miR-15a-5p; hsa-miR-21-5p; hsa-miR-128-3p; hsa-miR-493-3p; hsa-miR-146a-5p; hsa-miR-24-3p; hsa-miR-27b-3p |
| Notch signaling involved in heart development | ↑ | 3.81e-5 | 0.010 | hsa-miR-410-3p; hsa-miR-9-3p; hsa-miR-128-3p; hsa-miR-146a-5p; hsa-miR-30c-5p; hsa-miR-27b-3p |
| activation of protein kinase activity | ↑ | 9.93e-5 | 0.021 | hsa-miR-410-3p; hsa-miR-9-3p; hsa-miR-576-5p; hsa-miR-101-3p; hsa-miR-21-5p; hsa-miR-128-3p; hsa-miR-146a-5p; hsa-miR-24-3p |

**Supplementary Table 5**. Significant enrichment of inflammatory pathways. Top 10 over-represented reactome pathways. ↑ over-represented

| **Reactome (miRPathDB)** | **Enrichment** | **P-value** | **P-adjusted** | **miRNAs/precursors** |
|---|---|---|---|---|
| Diseases associated with the TLR signaling cascade | ↑ | 8.95e-4 | 0.024 | hsa-miR-146b-5p; hsa-miR-146a-5p |
| Diseases of Immune System | ↑ | 8.95e-4 | 0.024 | hsa-miR-146b-5p; hsa-miR-146a-5p |
| Interleukin-10 signaling | ↑ | 2.45e-4 | 0.024 | hsa-miR-21-5p; hsa-miR-146a-5p; hsa-miR-24-3p |
| Interleukin-17 signaling | ↑ | 3.59e-4 | 0.024 | hsa-miR-9-3p; hsa-miR-15a-5p; hsa-miR-21-5p; hsa-miR-493-3p; hsa-miR-146b-5p; hsa-miR-146a-5p |
| Interleukin-36 pathway | ↑ | 8.95e-4 | 0.024 | hsa-miR-146b-5p; hsa-miR-146a-5p |
| MAP kinase activation | ↑ | 4.24e-4 | 0.024 | hsa-miR-9-3p; hsa-miR-15a-5p; hsa-miR-21-5p; hsa-miR-493-3p; hsa-miR-146b-5p; hsa-miR-146a-5p |
| MyD88-independent TLR4 cascade | ↑ | 3.74e-4 | 0.024 | hsa-miR-9-3p; hsa-miR-301a-3p; hsa-miR-15a-5p; hsa-miR-21-5p; hsa-miR-493-3p; hsa-miR-146b-5p; hsa-miR-146a-5p |
| MyD88MALTIRAP cascade initiated on plasma membrane | ↑ | 7.80e-4 | 0.024 | hsa-miR-9-3p; hsa-miR-15a-5p; hsa-miR-21-5p; hsa-miR-493-3p; hsa-miR-146b-5p; hsa-miR-146a-5p |

**UPDATED August 2024**

| | | | | |
|---|---|---|---|---|
| MyD88 cascade initiated on plasma membrane | ↑ | 8.98e-4 | 0.024 | hsa-miR-9-3p; hsa-miR-15a-5p; hsa-miR-21-5p; hsa-miR-493-3p; hsa-miR-146b-5p; hsa-miR-146a-5p |
| MyD88 dependent cascade initiated on endosome | ↑ | 7.80e-4 | 0.024 | hsa-miR-9-3p; hsa-miR-15a-5p; hsa-miR-21-5p; hsa-miR-493-3p; hsa-miR-146b-5p; hsa-miR-146a-5p |

**Supplementary Table 6.** Embryo quality per patient sample, implantation, and live birth rates in fresh and cryopreserved single embryo transfers within one ovarian stimulation cycle, stratified by cluster.

| **Cluster** | **Embryo quality /sample** | **Implantation rate (FET %)** | **Implantation rate (CET %)** | **Live birth rate (FET, CET %)** |
|---|---|---|---|---|
| 1 | 5 (G), 28 (G), 33 (F), 72 (NB), 81 (P, F) | 0 | 50 | 20 |
| 2 | 11 (F), 29 (G), 46 (NB), 75 (G) | 66.67 | - | 66.67 |
| 3 | 13 (G), 31 (NB), 36 (NB), 42 (G), 77 (E), 92 (E, G, E) | 66.67 | 66.67 | 66.67 |

Abbreviations: excellent (E); good (G); fair (F); poor (P); non blast (NB); fresh embryo transfer (FET); cryo embryo transfer (CET)

**UPDATED August 2024**
